# A review and meta-analysis of prospective studies of red and processed meat intake and prostate cancer

**DOI:** 10.1186/1475-2891-9-50

**Published:** 2010-11-02

**Authors:** Dominik D Alexander, Pamela J Mink, Colleen A Cushing, Bonnie Sceurman

**Affiliations:** 1 Health Sciences Practice, Exponent Inc.; 185 Hansen Court, Suite 100, Wood Dale, IL 60191, USA; 2 Department of Epidemiology, Rollins School of Public Health, Emory University; 1518 Clifton Road, Room 3031 CNR Bldg, Atlanta, GA 30322, USA; 3 Health Sciences Practice, Exponent Inc.; 1150 Connecticut Ave NW, Washington D.C, USA

## Abstract

Over the past decade, several large epidemiologic investigations of meat intake and prostate cancer have been published. Therefore, a meta-analysis of prospective studies was conducted to estimate potential associations between red or processed meat intake and prostate cancer. Fifteen studies of red meat and 11 studies of processed meat were included in the analyses. High vs. low intake and dose-response analyses were conducted using random effects models to generate summary relative risk estimates (SRRE). No association between high vs. low red meat consumption (SRRE = 1.00, 95% CI: 0.96-1.05) or each 100 g increment of red meat (SRRE = 1.00, 95% CI: 0.95-1.05) and total prostate cancer was observed. Similarly, no association with red meat was observed for advanced prostate cancer (SRRE = 1.01, 95% CI: 0.94-1.09). A weakly elevated summary association between processed meat and total prostate cancer was found (SRRE = 1.05, 95% CI: 0.99-1.12), although heterogeneity was present, the association was attenuated in a sub-group analysis of studies that adjusted for multiple potential confounding factors, and publication bias likely affected the summary effect. In conclusion, the results of this meta-analysis are not supportive of an independent positive association between red or processed meat intake and prostate cancer.

## Introduction

Worldwide, prostate cancer is the second most common cancer among men, with only lung cancer accounting for more cancer diagnoses annually, although the incidence of prostate cancer varies considerably by geographic region [1]. Indeed, adopting a "Western" lifestyle has been hypothesized as contributing to the geographic variation in incidence rates. Studies of populations migrating to the United States (U.S.) from Japan and China have shown that the rate of prostate cancer increased compared to those in their native countries, independent of early detection [2-5], suggesting that lifestyle and dietary habits may contribute to the increasing rates of disease [6-11]. In the U.S., prostate cancer is the most commonly diagnosed cancer among men, accounting for approximately one-quarter of all new cancer cases, and this malignancy ranks as the second most common cause of cancer mortality, particularly among black males [12].

Although diet and prostate cancer has been investigated in numerous epidemiologic studies, few foods have been identified as potentially contributing to increasing or decreasing the risk of this malignancy. High intake of foods containing lycopene and selenium may decrease risk of prostate cancer while diets high in calcium may increase risk [3,13], although the epidemiologic evidence is not entirely clear. Some early studies have suggested that fat intake may be associated positively with prostate cancer [7,14,15], but recent prospective studies have reported no associations with fat consumption [16,17].

Over the last decade, several large cohort studies of meat intake and prostate cancer have been published, and in a recent systematic review of dietary factors, it was suggested that high meat consumption may increase the risk of prostate cancer, although the authors did not quantify the relationship [3]. In their 2007 report on diet and cancer, the World Cancer Research Fund/American Institute for Cancer Research concluded that there was "limited evidence from sparse and inconsistent studies suggesting that processed meat is a cause of prostate cancer (pg. 124)," however, their assessment was based on only four cohort studies [13]. Their judgment for other types of meat was "limited-no conclusion" [13]. Therefore, to explore further the potential relation between meat intake and prostate cancer, we conducted a meta-analysis of prospective studies to 1) estimate the summary associations between red meat and processed meat and total prostate cancer, 2) evaluate associations among men with advanced disease, 3) estimate dose-response trends, 4) evaluate potential sources of heterogeneity, and 5) assess the potential for publication bias.

## Materials and methods

A PubMed search of articles published through January, 2009, was conducted to identify studies eligible for review. The primary search string included the following terms: *prostate cancer *AND (*meat *OR *beef *OR *pork *OR *lamb*), yielding 143 articles. A supplemental search was conducted using *fat *AND *prostate cancer*, which yielded 482 additional articles. In addition, the bibliographies of review articles pertaining to diet and prostate cancer were examined in an effort to identify all available literature that may not have been identified by the PubMed search. Prospective epidemiologic studies, published in the English language, that reported results for the association between red meat or processed meat consumption and prostate cancer were included in this meta-analysis. Case-control studies, ecologic assessments, correlation studies, and other publications of aggregate-level analyses were excluded, as were experimental animal studies and mechanistic studies. Studies that reported only data for a broad classification of meat, such as 'total meat' categories, which included poultry or fish, were excluded. Studies were required to report point estimates (i.e., relative risks) and measures of variability (i.e., 95% confidence intervals) for a high category of red or processed meat intake compared with the lowest category of intake, or data were required to be available for such calculations.

Qualitative information and quantitative data were extracted from each study that met the criteria for inclusion. Specifically, information was extracted pertaining to: the year of the study, the study population (i.e., name and nature of the cohort), geographic location of the study, years of follow-up, methods of dietary exposure ascertainment, red meat and processed meat dietary variables and how these variables were defined, the analytical comparison (i.e., the exposure contrast), the number of exposed cases, the relative risk estimates and 95% confidence intervals, and the factors that were adjusted or controlled for in the analyses. A thorough review of each article was conducted to identify cohorts that may have been analyzed in multiple publications. If results were reported in multiple publications, the inclusion of data was based on 1) the size of the study population, 2) duration of follow-up with an emphasis on the most recent publication with the longest follow-up, 3) classification and analytical categorization of red or processed meat, and 4) level of control for potential confounding factors.

Random effects models were used to calculate summary relative risk estimates (SRRE), 95% confidence intervals, and corresponding p-values for heterogeneity. This type of model assumes that the study-specific effect sizes come from a random distribution of effect sizes according to a specific mean and variance [18]. Red meat is commonly defined as beef, pork, lamb, or a combination thereof [13,19], and processed meat is generally defined as meat made largely from pork, beef, or poultry that undergoes methods of preservation, such as curing, smoking, or drying [13,19]. The definitions of red meat and processed meat varied across studies; while some studies explicitly defined these classifications other studies reported no description. Most studies reported data for variables labeled as 'red meat,' 'processed' or 'preserved' meat, although some studies reported data for single meat items, such as beef, pork, liver, or bacon. Meta-analysis models were created for high vs. low red meat and processed meat intake. In addition to high vs. low intake analyses, categorical dose-response regression meta-analyses were conducted using the method proposed by Greenland and Longnecker [20], in which the linear dose-response slope is calculated for each study while accounting for the correlation across intake categories within a study [21]. If the number of cases and person-time data were not available for each intake strata, variance weighted least squares regression was utilized to estimate the slope coefficient. Different intake units were reported across studies; therefore, we used 100 grams as the approximate serving size for red meat and 50 grams for processed meat. Sensitivity analyses were conducted for a variety of methodological quality factors, such as level of adjustment for confounding factors, specification of red/processed meat variable, and number of food items ascertained in the FFQ.

Publication bias was assessed by generating funnel plots for a visual examination, conducting correlation and regression tests for significance, and using a 'trim and fill' procedure to evaluate symmetry around the summary effect [18]. All analyses were performed using STATA [22] and Comprehensive Meta-Analysis [23].

### Summary of cohort studies of red meat and prostate cancer

The characteristics and findings of the prospective studies of red and processed meat and prostate cancer are summarized below and in Table 1.

**Table 1:** Summary of cohort studies of red meat or processed meat and prostate cancer

Author and Year	Cohort	Exposure Variable (Definition)	Number of Exposed Cases	Analytical Comparison	**Relative Risk Estimate**^**a **^**(95% CI)**	Statistical Adjustment
Allen et al. 2008	European Prospective Investigation into Cancer and Nutrition (EPIC)	Red meat	371	5^th ^quintile vs. 1 (median intake = 90 g/day)	0.96 (0.82-1.12)	Stratified by center and adjusted for education, marital status, height, weight and energy intake
		Processed meat	590	5^th ^quintile vs. 1 (median intake = 88 g/day)	0.93 (0.79-1.09)	
Allen et al. 2004	Hiroshima and Nagasaki, Japan	Pork	8	Almost daily vs. <2 times/wk	1.24 (0.61-2.54)	Age, calendar period, city of residence, radiation dose, and education level
Chan et al. 2000	ATBC Study (Finland)	Red meat	NR	Quintiles of intake: 5 vs. 1	0.7 (0.5-1.1)	Supplementation, education, and quintiles of age, BMI, energy and smoking
Cross et al. 2007	NIH-AARP Diet & Health Study	Red meat (all types of beef, pork, and lamb; including bacon, beef, cold cuts, ham, hamburger, hot dogs, liver, pork, sausage, and steak; meats added to mixtures, such as pizza, chili, lasagna, and stew)		Quintiles of intake: 5 vs. 1 62.7 g/1000 kcal vs. 9.8		Age, sex, education, marital status, family hx of cancer, race, BMI, smoking, frequency of vigorous physical activity, total energy intake, alcohol intake, and fruit and vegetable consumption
			3,950	All cases	1.01 (0.96-1.07)	
			NR	Advanced cases	1.15 (0.98-1.36)	
		Processed meat (bacon, red meat sausage, poultry sausage, luncheon meats, cold cuts, ham, hot dogs, meats added to mixtures, such as pizza, chili, lasagna, and stew)		Quintiles of intake: 5 vs. 1 22.6 g/1000 kcal vs. 1.6		
			4,196	All cases	1.02 (0.97-1.07)	
			NR	Advanced cases	1.22 (1.05-1.43)	
Cross et al. 2005	PLCO Cancer Screening Trial	Red meat (all beef, pork, and lamb [processed & non-processed])		Quintiles of intake: 5 vs. 1		Age, race, study center, family hx of prostate cancer, hx of diabetes, number of screening exams during follow-up, smoking status, physical activity, aspirin use, BMI, and intake of total energy, supplemental vitamin E, lycopene
			NR	All cases	0.91 (0.73-1.12)	
			NR	Incident cases	0.81 (0.62-1.06)	
			NR	Advanced cases	0.92 (0.66-1.29)	
		Processed meat (Ham, hot dogs, liver, cold cuts, sausage, bacon)		Quintiles of intake 5 vs. 1		
			NR	All cases	1.14 (0.93-1.39)	
			NR	Incident cases	1.16 (0.91-1.50)	
			NR	Advanced cases	1.37 (0.99-1.90)	
Gann et al. 1994 *	Physician's Health Study	Beef, pork or lamb as a main dish	NR	Consumption: ≥5-6 times/wk vs. ≤1-3 times/month	2.51 (0.93-6.74)	Matched by age and smoking status
Hsing et al. 1990	Lutheran Brotherhood Society Cohort	Meat (beef, bacon, fresh pork, and smoked ham)	Mortalities	Intake (times/month)		Age and tobacco use
			27	>39 vs. <17	0.8 (0.5-1.3)	
Koutros et al. 2008	Agricultural Health Study (Iowa and North Carolina)			Quintiles of intake: 5 vs. 1		Adjusted for age, state of residence, race, family hx of prostate cancer, and smoking status
		Red meat	105	All cases	1.10 (0.85-1.43)	
			95	Incident cases	1.11 (0.84-1.46)	
			21	Advanced cases	0.89 (0.50-1.60)	
		Bacon/sausage	140	All cases	0.98 (0.78-1.24)	
			125	Incident cases	0.90 (0.70-1.15)	
			21	Advanced cases	0.69 (0.40-1.18)	
Le Marchand et al. 1994	Hawaii	Pork	NR	Quantile of intake 4 vs. 1 (range for cohort 0-118 g/wk)	1.1 (0.7-1.7)	Age, ethnicity, and income
		Beef		Tertile of intake 3 vs. 1 (range for cohort 210-381 g/wk)		Age, ethnicity, and income by proportional hazards regression
			NR	All cases	1.6 (1.1-2.4)	
			NR	Diagnosis age ≤72.5 yrs	2.2 (1.2-4.1)	
			NR	Diagnosis age >72.5 yrs	1.4 (0.8-2.5)	
			NR	Diagnosis age ≤72.5 yrs, localized stage prostate cancer	2.7 (NR)	
			NR	Diagnosis age >72.5 yrs, localized stage prostate cancer	2.0 (NR)	
			NR	Diagnosis age ≤72.5 yrs, regional and distant stage prostate cancer	1.4 (NR)	
			NR	Diagnosis age >72.5 yrs, regional and distant stage prostate cancer	0.8 (NR)	
		Processed meat	NR	Quantiles of intake 4 vs. 1(range for cohort 0-129 g/wk)	1.2 (0.8-1.9)	
Michaud et al. 2001	Health Professionals Follow-Up Study	Red meat (processed meats; bacon; hot dogs; hamburger; beef, pork, or lamb as a sandwich, mixed dish or main dish)		Quintiles 5 vs. 1	0.91 (0.75-1.1)	Age, calories, calcium, smoking, tomato sauce, vigorous exercise, saturated fat and alpha-linolenic fat
			315	Prostate cancer excluding stage A1		
			104	Advanced prostate cancer	1.15 (0.80-1.7)	
			55	Metastatic prostate cancer	1.50 (0.88-2.5)	Also adjusted for period, in addition to covariates above
		Beef, pork or lamb (main dish)	38	Intake of 5+ vs. 0/wk, metastatic prostate cancer	1.35 (0.72-2.5)	Age, calories, calcium, smoking, tomato sauce, vigorous exercise, saturated and alpha linolenic fat
				Intake of 2+ vs. 0/wk,		
		Beef, pork or lamb (sandwich or mixed dish)	64	Metastatic prostate cancer	0.96 (0.62-1.5)	
		Hamburger	68	Metastatic prostate cancer	1.08 (0.66-1.8)	
		Processed meats	71	Metastatic prostate cancer	1.39 (0.94-2.1)	Age, calories, calcium, smoking, tomato sauce, vigorous exercise, saturated fat, and alpha-linolenic fat
		Bacon	50	Metastatic prostate cancer	1.33 (0.89-2.0)	
		Hot dogs	15	Metastatic prostate cancer	0.85 (0.48-1.5)	
Mills et al. 1989	Seventh Day Adventists	Beef hamburger	43	Consumed ≥1 time/wk vs. never	1.07 (0.73-1.59)	Age
		Beef steak	17		0.81 (0.72-1.50)	
		Other beef and veal	32		1.09 (0.71-1.67)	
		Beef index	63		1.21 (0.83-1.75)	
Neuhouser et al. 2007	CARET	Red meat		Quartiles of intake: high vs. low		Age, energy intake, BMI, smoking, family hx of prostate cancer
			NR	Prostate cancer	0.76-1.62§ (NR)	
Park et al. 2007	Multiethnic Cohort Study			Quintile of intake: 5 vs. 1		
		Red meat (beef, pork, and lamb)	NR	Total prostate cancer	0.97 (0.87-1.07)	Time on study, ethnicity, family hx of prostate cancer, education, BMI, smoking status, energy intake
			NR	Nonlocalized or high-grade cancer	0.95 (0.79-1.14)	
			226	African Americans	1.05 (0.86-1.27)	Time on study, family hx of prostate cancer, education, BMI, smoking status, energy intake
			109	Japanese Americans	1.04 (0.82-1.31)	
			270	Latinos	0.87 (0.72-1.06)	
			115	Whites	0.83 (0.65-1.05)	
		Beef	NR	Total prostate cancer	0.98 (0.88-1.08)	Time on study, ethnicity, family hx of prostate cancer, education, BMI, smoking status, energy intake
			NR	Nonlocalized or high-grade cancer	0.97 (0.81-1.16)	
		Pork	NR	Total prostate cancer	0.97 (0.88-1.08)	
			NR	Nonlocalized or high-grade cancer	0.92 (0.76-1.11)	
		Processed meat (processed red meat and processed poultry)		Quintile of intake: 5 vs. 1		
			NR	Total prostate cancer	1.01 (0.91-1.12)	Time on study, ethnicity, family hx of prostate cancer, education, BMI, smoking status, energy intake
			NR	Nonlocalized or high-grade cancer	0.92 (0.77-1.11)	
			373	African Americans	1.00 (0.83-1.20)	Time on study, family hx of prostate cancer, education, BMI, smoking status, energy intake
			181	Japanese Americans	1.09 (0.88-1.34)	
			134	Latinos	0.86 (0.69-1.08)	
			157	Whites	1.02 (0.82-1.27)	
Rodriguez et al. 2006	Cancer Prevention Study II	Total processed plus unprocessed red meat (includes both processed meat and red meat)		Intake: ≥657 vs. <246 g/wk		Age at entry, total calorie intake, BMI, education, family hx of prostate cancer, hx of PSA testing, and hx of diabetes
			27	All prostate cancer, Blacks	2.0 (1.0-4.2)	
			1,239	All prostate cancer, Whites	1.0 (0.9-1.0)	
			56	Metastatic prostate cancer, Whites	0.8 (0.5-1.3)	
		Unprocessed red meat		Intake: ≥423 vs. <137 g/wk		
			20	All prostate cancer, Blacks	1.7 (0.8-3.9)	
			1,557	All prostate cancer, Whites	1.0 (0.9-1.1)	
			69	Metastatic prostate cancer, Whites	0.8 (0.5-1.2)	
		Processed meats (includes both cooked processed meat and lunchmeat)		Intake ≥247 vs. <59 g/wk		
			28	All prostate cancer, Blacks	2.4 (1.2-4.9)	
			765	All prostate cancer, Whites	1.0 (0.9-1.1)	
			37	Metastatic prostate cancer, Whites	1.1 (0.7-1.7)	
		Cooked processed meat		Intake ≥165 vs. <38 g/wk		
			29	All prostate cancer, Blacks	2.7 (1.3-5.3)	
			369	All prostate cancer, Whites	1.0 (0.9-1.2)	
			21	Metastatic prostate cancer, Whites	1.2 (0.7-2.1)	
		Lunchmeat		Intake ≥56 g/wk vs. none		
			29	All prostate cancer, Blacks	1.0 (0.6-1.9)	
			1,845	All prostate cancer, Whites	1.0 (1.0-1.1)	
			88	Metastatic prostate cancer, Whites	1.0 (0.7-1.5)	
Rohrmann et al. 2007	CLUE II	Red meat (hamburgers, beef, beef stew, pork, hot dogs, ham/lunch meats, bacon, sausages)		Tertile of daily consumption (3 vs. 1)		Age, energy intake, consumption of tomato products, BMI at age 21, and intake of saturated fat
			51	Total prostate cancer	0.87 (0.59-1.32)	
			12	High-stage prostate cancer	0.87 (0.39-1.93)	
			17	Low-stage prostate cancer	0.60 (0.31-1.18)	
		Beef (beef, beef stew, pork, hot dogs, ham/lunch meats, bacon, sausages)		Consumption: >5 vs. ≤1 times/wk		
			84	Total prostate cancer	1.16 (0.74-1.81)	
			18	High-stage prostate cancer	0.83 (0.36-1.92)	
			35	Low-stage prostate cancer	1.72 (0.79-3.79)	
		Pork		Consumption: ≥1 times/wk vs. never		
			39	Total prostate cancer	1.17 (0.77-1.78)	
			12	High-stage prostate cancer	1.98 (0.87-4.53)	
			15	Low-stage prostate cancer	0.88 (0.46-1.70)	
		Processed meats		Consumption : >5 vs. ≤1 times/wk		
			96	Total prostate cancer	1.53 (0.98-2.39)	
			27	High-stage prostate cancer	2.24 (0.90-5.59)	
			32	Low-stage prostate cancer	1.30 (0.62-2.74)	
				Consumption : ≥1 vs. <1 times/wk		
		Sausages	43	Total prostate cancer	1.16 (0.79-1.73)	Age, energy intake, saturated fat intake, consumption of tomato products, and BMI at age 21
			17	High-stage prostate cancer	2.83 (1.34-5.99)	
			14	Low-stage prostate cancer	0.75 (0.39-1.44)	
		Bacon	74	Total prostate cancer	1.32 (0.91-1.93)	
			22	High-stage prostate cancer	2.10 (0.97-4.53)	
			31	Low-stage prostate cancer	1.35 (0.74-2.44)	
		Ham/lunch meat	115	Total prostate cancer	1.54 (1.01-2.33)	
			30	High-stage prostate cancer	1.94 (0.82-4.56)	
			43	Low-stage prostate cancer	2.00 (0.94-4.25)	
		Hot dogs	45	Total prostate cancer	1.12 (0.73-1.73)	
			13	High-stage prostate cancer	1.71 (0.70-4.14)	
			18	Low-stage prostate cancer	0.96 (0.50-1.88)	
Schuurman et al. 1999**	Netherlands Cohort Study			Continuous variables of 25 g/day increments		Age, family hx of prostate cancer, socioeconomic status, total fresh meat and poultry
		Beef	NR	All tumors	1.00 (0.89-1.12)	
			NR	Localized tumors	0.95 (0.80-1.12)	
			NR	Advanced tumors	0.92 (0.77-1.10)	
		Pork	NR	All tumors	1.06 (0.96-1.18)	
			NR	Localized tumors	1.16 (1.00-1.34)	
			NR	Advanced tumors	1.06 (0.91-1.23)	
		Minced meat (beef and pork)	NR	All tumors	0.86 (0.74-1.01)	
			NR	Localized tumors	0.84 (0.66-1.07)	
			NR	Advanced tumors	0.90 (0.71-1.14)	
				Continuous variables of 5 g/day increments		
		Liver	NR	All tumors	0.92 (0.82-1.04)	
			NR	Localized tumors	0.99 (0.85-1.17)	
			NR	Advanced tumors	0.79 (0.63-0.99)	
		Other meat (horsemeat, lamb, mutton, and veal)	NR	All tumors	1.06 (0.99-1.15)	
			NR	Localized tumors	1.04 (0.93-1.16)	
			NR	Advanced tumors	1.09 (0.98-1.21)	
		Cured meat (boiled ham, bacon. Lean meat products including smoked beef, and other sliced cold meats)	123	Quintiles of intake 5 vs. 1 36 g/day vs. 0	1.37 (1.00-1.89)	Age, family hx of prostate cancer and socioeconomic status
			NR	Continuous variables of 15 g/day increments Advanced tumors	1.00 (0.88-1.14)	
Severson et al. 1989	Hawaii	Ham, bacon, sausage	35	≥5/vs. ≤1 times/wk	1.11 (0.75-1.65)	Age
Wu et al. 2006	US Health Professionals	Total red meat (hamburger, beef, lamb, and pork as main dish; beef, lamb, & pork as main dish or mixed dish)		Intake quintile 5 vs. 1		Age, height, smoking, family hx of prostate cancer, race, hx of vasectomy, vigorous exercise, BMI, alcohol intake, and total energy intake
			41	Age <65 yrs old	2.12 (1.18-3.78)	
			72	Age ≥65 yrs old	1.21 (0.85-1.74)	
		Processed meats (salami, bologna, or other processed meat sandwiches; sausage, kielbasa, hot dogs, and bacon)		Intake quintile 5 vs. 1		
			34	Age <65 years old	0.85 (0.47-1.56)	
			79	Age ≥65 years old	1.51 (1.00-2.26)	

In a recent analysis of the Agricultural Health Study, which followed a cohort of 57,311 licensed pesticide applicators from Iowa and North Carolina, Koutros et al. [24] reported a non-significant positive association between red meat intake and prostate cancer (RR = 1.10; 95% CI: 0.85-1.43), and a non-significant inverse association for advanced prostate cancer was observed (RR = 0.89; 95% CI: 0.50-1.60). In their analysis of processed meat (i.e., bacon and sausage consumption), associations of 0.98 (95% CI: 0.78-1.24) and 0.69 (95% CI: 0.40-1.18) were observed for total prostate cancer and advanced prostate cancer, respectively.

In the largest study of red/processed meat and prostate cancer to date, Cross and colleagues [25] analyzed over 17,000 cases of prostate cancer from the National Institutes of Health (NIH)-AARP (formerly the American Association for Retired Persons) Diet and Health Study and observed no association between the highest levels of red meat (RR = 1.01, 95% CI: 0.96-1.07) or processed meat (RR = 1.02, 95% CI: 0.97-1.07) intake and total prostate cancer. Although the risks were elevated slightly for red meat (RR = 1.15, 95% CI = 0.98-1.36) and processed meat intake (RR = 1.22, 95% CI = 1.05-1.43) and advanced prostate cancer, the tests for trend were not significant. In a recent sub-group analysis of this cohort that focused on meat compounds and cooking methods, positive associations were reported for red meat and total (RR = 1.12, 95% CI: 1.04-1.21), advanced (RR = 1.31, 95% CI: 1.05-1.65), and fatal (RR = 1.25, 95% CI: 0.87-1.82) prostate cancer [26]. Similar associations were observed for processed meat intake and total (RR = 1.07, 95% CI: 1.00-1.14) and advanced (RR = 1.32, 95% CI: 1.08-1.61) prostate cancer, although an inverse associations was reported for fatal prostate cancer (RR = 0.86, 95% CI: 0.63-1.18) [26].

In an analysis of the prospective American Cancer Society's (ACS) Cancer Prevention Study II (CPS II) Nutrition Cohort, Rodriguez et al. [27] reported no significant associations (RRs ranged between 0.7 and 1.1) between unprocessed red meat intake or total processed plus unprocessed red meat intake and total prostate cancer and metastatic prostate cancer among white men (5,028 cases). Similarly, non-significant associations (RR range: 0.9-1.3) between processed meat, cooked processed meat, luncheon meat and total and metastatic prostate cancer were reported among white men. Associations for red and processed meat were stronger among black men; however, analyses were limited to fewer than 30 cases at the highest meat intake categories. Among black men, the RR for the highest category of total processed plus unprocessed red meat intake was 2.0 (95% CI: 1.0-4.2) while the RR for unprocessed red meat was 1.7 (95% CI: 0.8-3.9). Associations were elevated significantly for the highest intake categories of processed meat (RR = 2.4; 95% CI: 1.2-4.9) and cooked processed meat (RR = 2.7; 95% CI: 1.3-5.3), but not lunchmeat (RR = 1.0; 95% CI: 0.6-1.9). Because of the small number of cases, only total prostate cancer was analyzed for black men.

In an analysis of over 1,300 prostate cancer cases in the multi-center Prostate, Lung, Colorectal, and Ovarian (PLCO) Cancer Screening Trial, RRs comparing the highest vs. lowest intake levels of red meat were below 1.0 and non-significant for all prostate cancer cases, incident cases only, and advanced cases only (RRs = 0.91, 0.81, and 0.92, respectively) [28]. Associations between processed meat and all prostate cancer and incident prostate cancer were similar, and were slightly but not significantly elevated (1.14 and 1.16, respectively). For advanced prostate cancer, the RR for the highest versus lowest quintile was 1.37 (95% CI: 0.99-1.90), although the test for trend was not significant (p-trend = 0.32).

In their analyses of data from the Health Professionals Follow-Up Study, Michaud et al. [29] observed a non-significant inverse association between red meat intake and total prostate cancer (RR for highest versus lowest quintile = 0.91; 95% CI: 0.75-1.1). In contrast, non-significant positive associations were reported for advanced (RR = 1.15) and metastatic (RR = 1.50) prostate cancer, but no significant trends were observed. Moreover, the authors noted that while the risk for metastatic prostate cancer was elevated, the association with red meat was attenuated after adjusting for saturated and α-linolenic fatty acids in addition to the other covariates in the model. Among metastatic cases, the RRs for the highest intake categories of processed meat, bacon, and hot dogs were 1.39 (95% CI: 0.94-2.1), 1.33 (95% CI: 0.89-2.0), and 0.85 (95% CI: 0.48-1.5), respectively. In a subsequent publication of this cohort, Wu et al. [30] evaluated dietary patterns and prostate cancer, although the authors reported data specifically for red and processed meat by age strata. Among men < 65 years of age, the RR for total red meat intake was 2.12 (95% CI: 1.18-3.78), and the RR for men 65 and older was 1.21(95% CI: 0.85-1.74) after adjustment for a western dietary pattern and other covariates. In contrast to the analysis of red meat, the association for processed meat was stronger among men 65 and older (RR = 1.51, 95% CI: 1.00-2.26) compared with men younger than age 65 (RR = 0.85, 95% CI: 0.47-1.56).

Park et al. [17] examined the association between meat and fat intake and prostate cancer risk in the Multiethnic Cohort Study, which included over 80,000 men in Hawaii and Los Angeles. Inverse associations were observed for red meat intake and total prostate cancer (RR = 0.97, 95% CI: 0.87-1.07) and high-grade cancer (RR = 0.95, 95% CI: 0.79-1.14). Among Whites, Latinos, Japanese Americans, and African Americans, RRs were 0.83, 0.87, 1.04, and 1.05, respectively, and none of the associations were statistically significant. Similar associations were observed for processed meat intake, with RRs of 1.01 and 0.92 for total prostate cancer and high-grade cancer, respectively. Among ethnic groups, RRs for processed meat ranged between 0.86 and 1.09 and were not significant.

Allen et al. [31] evaluated animal foods, protein, and calcium among over 140,000 men in the European Prospective Investigation into Cancer and Nutrition (EPIC) cohort. Participants were followed 8.7 years, on average, and 2,727 men were diagnosed with prostate cancer. The authors observed decreased risks of total prostate cancer among men in the highest consumption categories of red (HR = 0.96. 95% CI: 0.82-1.12; median intake in highest category = 90 g/day) and processed meat (HR = 0.93, 95% CI: 0.79-1.09; median intake in highest category = 88 g/day) after adjustment for education, marital status, height, weight and energy intake.

In the Netherlands Cohort Study (NLCS), Schuurman et al. [32] reported non-significant RRs for total prostate cancer of 1.0, 1.06, 0.86, and 0.92 for the highest intake categories of beef, pork, minced meat (beef and pork), and liver, respectively. The RR for pork and localized prostate tumors was marginally significant (RR = 1.16; 95% CI: 1.00-1.34), whereas the RR for liver and advanced prostate tumors was inverse and statistically significant (RR = 0.79; 95% CI: 0.63-0.99). The RRs for increasing quintiles of cured meat were 1.0 (referent group: no consumption), 1.22, 1.50, 1.18, and 1.37, respectively (p-trend = 0.04).

In a modestly sized study of almost 4,000 participants from the CLUE II cohort, red and processed meat intake was analyzed for approximately 200 cases of prostate cancer [33]. Multivariate-adjusted RRs for red meat intake and total prostate cancer, high-stage prostate cancer, and low-stage prostate cancer were below 1.0 and not statistically significant. Conversely, non-significant positive associations were reported for processed meat intake, with the strongest association found among persons with high-stage cancer (RR = 2.24) although this result was based on only 27 cases. Positive associations for total prostate cancer and high-stage prostate cancer were reported for individual processed meat items (i.e., sausages, bacon, ham/lunch meat, hot dogs) with two associations being statistically significant (RR for sausages and high-stage prostate cancer = 2.83, 95% CI: 1.34-5.99; RR for ham/lunch meat and total prostate cancer = 1.54. 95% CI: 1.01-2.33).

Chan et al. [34] evaluated associations between diet and incidence of clinical prostate cancer (stage 2-4 disease) among 27,111 Finnish participants in the Alpha-Tocopherol Beta-Carotene Cancer Prevention Study (ATBC Study). Although high levels of red meat intake were evaluated (i.e., median of 214 grams in highest intake category), all associations were inverse across the intake strata, with an RR of 0.7 (95% CI: 0.5-1.1) in the highest consumption category.

In a prospective study of diet and prostate cancer among Japanese men exposed to radiation during the bombings of Hiroshima or Nagasaki, the association between "almost daily" consumption of pork (versus < 2 times/week) was associated weakly and non-significantly with risk of prostate cancer (RR = 1.24; 95% CI: 0.61-2.54; p-trend = 0.14) [35]. No other red meat items were evaluated in this study.

In a nested case-control study conducted within the Physicians' Health Study cohort[36], the RR based on consuming beef, pork, or lamb as a main dish at least 5-6 times per week (versus 1-3 times per month or less) was 2.51 (95% CI: 0.93-6.74). This association is considerably higher than the associations reported in the other studies, although the confidence interval is wide, indicating imprecision in the estimate. The number of cases in the intake categories was not reported.

Le Marchand et al. [37], in a multi-ethnic, population-based prospective cohort study of diet and cancer in Hawaii reported no association for pork intake but observed a significantly elevated association for beef intake (RR = 1.6; 95% CI: 1.1-2.4). Associations were stronger among persons with localized disease compared with regional or distant stage cancer. A non-significant association for processed meat intake was observed (RR = 1.2, 95% CI: 0.8-1.9) but no trend was apparent (p-trend = 0.38). The authors used a 13 food-item questionnaire, thus, results may have been confounded by energy intake or other food items. In another study conducted in Hawaii [38], no significant association was observed for intake of ham, bacon, or sausage (5+ times per week vs. *<*1) (RR = 1.11, 95% CI: 0.75-1.65), although this result was adjusted for age only.

In a cohort of 17,633 white male Lutheran Brotherhood Insurance policy holders ("The Lutheran Brotherhood Cohort Study"), Hsing and colleagues [39] observed a non-significant inverse association between red/processed meat intake and prostate cancer mortality (RR = 0.8, 95% CI: 0.5-1.3). In an evaluation of participants in the Seventh Day Adventists cohort, Mills et al. [40] reported non-significant associations ranging between 0.81 and 1.21 for four beef intake variables.

### Summary of Meta-Analysis Results for Red and Processed Meat Intake and Prostate Cancer

**Figure 1:**
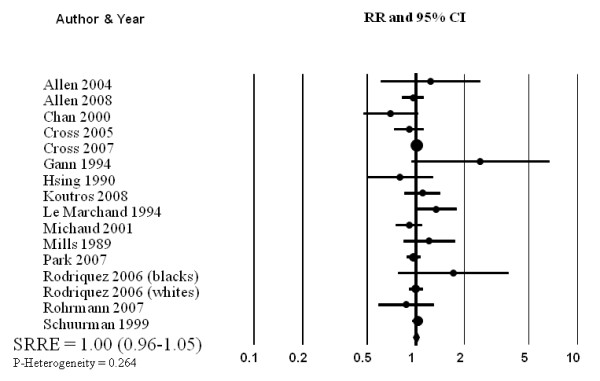
Meta-analysis of prospective studies of red meat intake and prostate cancer.

No association between consumption (high vs. low intake) of red meat and total prostate cancer was observed in the meta-analysis of 15 prospective studies (SRRE = 1.00, 95% CI: 0.96-1.05; p-value for heterogeneity = 0.264) (Table 2, Figure 1). The summary association changed slightly after excluding four studies that reported data for individual red meat items only (e.g., beef or pork) (SRRE = 0.98, 95% CI: 0.93-1.04). Modest effect modification was observed in the analyses by publication date; no association was found in the model restricted to the 10 published since 2000 (SRRE = 0.99, 95% CI: 0.95-1.03) while a weakly elevated summary association was observed in the studies published prior to 2000 (SRRE = 1.13, 95% CI: 0.92-1.37). Sensitivity analyses excluding a study that used a short food frequency questionnaire (13 items) [37] and an outlier study (greater than two-fold association) [36] did not alter the overall summary association (Table 2). Removing the non-U.S. studies did not modify the summary effect (SRRE = 1.00, 95% CI: 0.95-1.06, p-value for heterogeneity = 0.250). Similar to the high vs. low intake analysis, no association between each 100 g increment of red meat and prostate cancer was observed in the categorical dose-response regression meta-analysis (SRRE = 1.00, 95% CI: 0.95-1.05). Meta-analysis of eight studies of advanced prostate cancer resulted in an SRRE of 1.01 (95% CI: 0.94-1.09) with little heterogeneity (p for heterogeneity = 0.657). The SRRE for each 100 g increment of red meat and advanced prostate cancer was 0.97 (95% CI: 0.91-1.02), based on data from five studies that reported red meat as a food group variable.

**Table 2:** Summary of meta-analysis findings for red and processed meat intake and prostate cancer.

Model	# Studies	SRRE (95% CI)	P-Heterogeneity
***Red Meat***			

Total model (includes individual red meat items)	15	1.00 (0.96-1.05)	0.264

Red meat specific variable only ("red meat" as a food group)	11	0.98 (0.93-1.04)	0.353

Studies published during 2000-2009	10	0.99 (0.95-1.03)	0.593

Studies published prior to 2000	5	1.13 (0.92-1.37)	0.108

Studies that adjusted for at least three of the following factors: energy, smoking, family history of cancer, age, race	9	0.99 (0.95-1.03)	0.536

Le Marchand removed (13 food item questionnaire)	13	0.99 (0.95-1.03)	0.401

Gann removed (outlier study)	13	0.99 (0.95-1.04)	0.364

Advanced prostate cancer	8	1.01 (0.94-1.09)	0.657

100 g increment (total prostate cancer)*	9	1.00 (0.95-1.05)	0.007

100 g increment (advanced cancer)*	5	0.97 (0.91-1.02)	0.571

***Processed Meat***			

Total model	11	1.05 (0.99-1.12)	0.088

Michaud removed (data for metastatic prostate cancer only)	10	1.04 (0.98-1.11)	0.113

Studies published during 2000-2009 [Note: these are also the studies that adjusted for at least three of the following factors: energy, smoking, family history of cancer, age, race]	8	1.04 (0.97-1.11)	0.085

Studies published prior to 2000 [Note: these studies did not simultaneously adjust for three of the above factors]	3	1.25 (1.00-1.54)	0.705

Advanced prostate cancer	8	1.10 (0.95-1.27)	0.032

30 g increment (total prostate cancer)	10	1.02 (1.00-1.04)	0.274

30 g increment (advanced cancer)	6	1.01 (0.90-1.14)	0.020

*Includes studies that reported data for a "red meat" group variable

**Figure 2:**
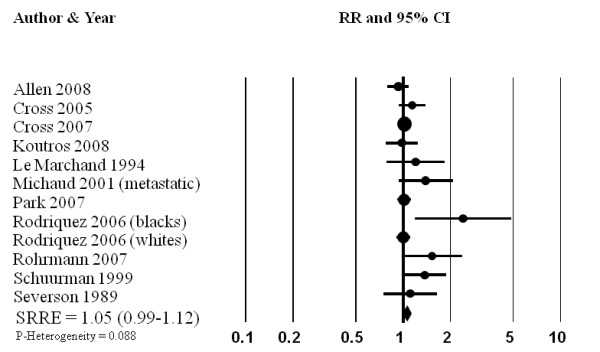
**Meta-analysis of prospective studies of processed meat intake and prostate cancer**.

The summary effect between processed meat and total prostate cancer was slightly elevated, although the estimates across studies were variable (SRRE = 1.05, 95% CI: 0.99-1.12; p for heterogeneity = 0.088) (Table 2, Figure 2). The summary association was modified by publication date and control for important confounding factors, as studies published between 2000-2009 and that adjusted simultaneously for energy intake, smoking, family history of cancer, age, or race (note: adjustment for at least 3 of these variables required for inclusion in this model) was 1.04 (95% CI: 0.97-1.11). The SRRE for the three studies published prior to 2000 and that did not adjust simultaneously for at least three of the aforementioned variables was 1.25 (95% CI: 1.00-1.54). Michaud et al. [29] reported data only for processed meat among metastatic cases; when this study was removed from the overall model, the SRRE became 1.04 (95% CI: 0.98-1.11). Removal of the single non-U.S. studies did not alter the summary effect although the model became more homogeneous (SRRE = 1.05, 95% CI: 0.98-1.12, p-value for heterogeneity = 0.157). In the categorical dose-response regression analysis, the SRRE for each 30 g increment of processed meat intake was 1.02 (95% CI: 1.00-1.04). No significant association between processed meat intake and advanced prostate cancer was found in the meta-analysis of eight studies (SRRE = 1.10, 95% CI; 0.95-1.27). The summary association among advanced cases was attenuated for each 30 g increment of processed meat intake (SRRE = 1.01, 95% CI: 0.90-1.14).

**Figure 3:**
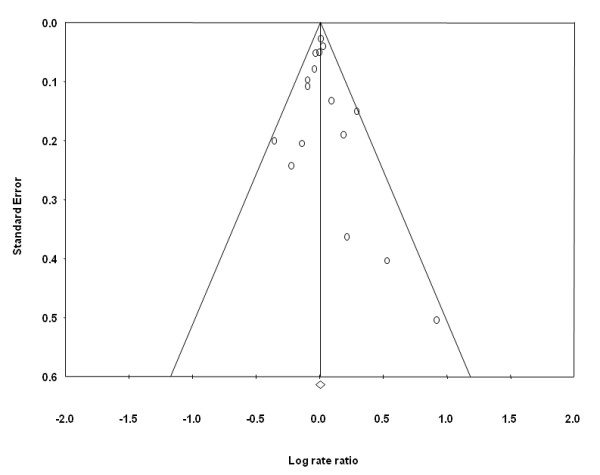
Funnel plot of prospective studies of red meat intake and prostate cancer.

**Figure 4:**
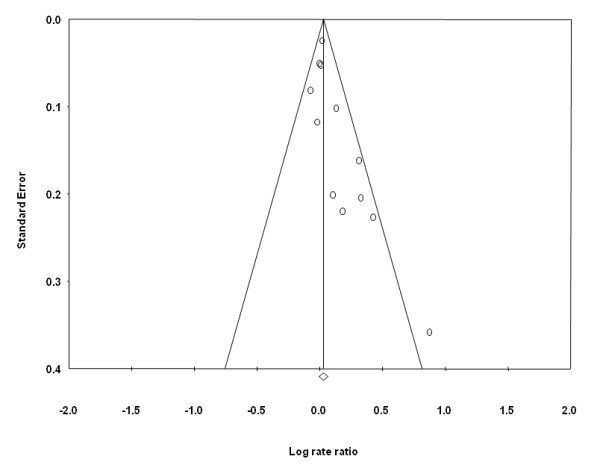
Funnel plot of prospective studies of processed meat intake and prostate cancer.

An assessment of the funnel plot of prospective studies of red meat and prostate cancer suggested slight publication bias (Figure 3), although statistical tests did not confirm this. Publication bias, however, was indicated in the funnel plot and statistical tests for processed meat. Point estimates from smaller studies, with greater variability, were more likely to be distributed on the positive side of the mean effect size (Figure 4). Furthermore, Egger's regression test was statistically significant (p = 0.013) and Duval and Tweedie's trim and fill procedure imputed four studies to the left of the mean effect, resulting in an adjusted SRRE of 1.02 (95% CI: 0.94-1.10) compared with 1.05 (95% CI: 0.99-1.12) from the overall model.

## Discussion

With the exception of increasing age, African-American race, and family history of prostate cancer, little is known about the etiology of prostate cancer [2]. Studies of persons migrating to westernized countries suggest that exogenous factors, such as adopting certain dietary and lifestyle characteristics, may contribute to increasing the risk of malignancy. As a result, diet has been the focus of numerous epidemiologic studies of prostate cancer, although findings have not been consistent. It has been suggested that red meat or processed meat may be responsible for increasing the risk of prostate cancer [2,3] but findings across the collective body of prospective cohort studies have not produced results indicative of an independent positive association. Therefore, we conducted a meta-analysis of prospective studies to clarify any potential relations between red meat or processed meat and prostate cancer.

The summary associations across the meta-analysis models of red meat intake ranged between 0.97 and 1.01, with the exception of one model (i.e., the SRRE was 1.13 for the five studies published prior to 2000), and none of the associations were statistically significant (Table 2). Furthermore, there was little heterogeneity across the red meat models. Analyses of high vs. low red meat intake and 100 g increment dose-response regression produced similar results; both SRREs were 1.0.

On average, summary associations for processed meat were slightly stronger in magnitude compared with red meat. However, greater heterogeneity was present in the analyses of processed meat. In sub-group analyses of the eight studies that adjusted simultaneously for at least three potentially important confounding factors, the SRRE was closer to the null compared with the three studies that did not adjust for these factors (1.04 vs. 1.25). Furthermore, the three studies that were not as fully-adjusted were published prior to the year 2000 and were not as large as the other studies. In addition, there was evidence of publication bias in the meta-analysis of processed meat. Theoretically, if all relevant studies were included in the meta-analysis, it would be expected that the funnel plot would be symmetric (i.e., even distribution of point estimates on either side of the mean effect), which was not the case for processed meat (Figure 4). If the funnel plot is asymmetric, and a higher number of smaller studies are dispersed on the right side of the summary effect, there may be additional small studies for which processed meat data was not reported [23]. Using the "trim and fill" method proposed by Duval and Tweedie, these potentially missing studies are imputed on the other side of the summary effect, and the overall summary association is recalculated. This method trims the asymmetric studies from the right-hand side to identify the unbiased effect (in an iterative procedure), and then fills the plot by re-inserting the trimmed studies on the right as well as their imputed counterparts to the left the mean effect [18,23]. In the analysis of processed meat, this method indicated that four studies may be missing. After imputing data from these potential studies, the SRRE changed from 1.05 (95% CI: 0.99-1.12) to 1.02 (95% CI: 0.94-1.10). It should be noted; however, that this method is merely estimating unpublished data, rather than relying upon actual data.

The summary association for processed meat and prostate cancer in the current assessment was relatively similar to the summary association reported in the aforementioned WCRF/AICR report on diet and cancer (summary RR per serving/week = 1.11, 95% CI: 0.99-1.25) [13]. However, their analysis included data from only four cohorts, whereas, in the current assessment, data from 11 prospective studies of processed meat were analyzed. Their analysis of case-control studies produced a summary effect estimate of 1.01 (95% CI 0.98-1.04) per each processed meat serving/week and prostate cancer [13]. WCRF/AICR judged that the epidemiologic evidence regarding processed meat intake and prostate cancer was "limited-suggestive" and was based on sparse and inconsistent data [13]. The epidemiologic data for red meat and prostate cancer were not summarized in their report.

Although an evaluation of correlates of meat consumption and prostate cancer is beyond the scope of the current assessment, a few factors thought to contribute to positive associations are worth mentioning. Few studies examined fat intake from animal sources, particularly red meat sources, and prostate cancer. Le Marchand et al. [37] reported that intake of "high fat animal products" was associated positively with prostate cancer (RR = 1.6, 95% CI: 1.1-2.4), although the source of animal fat was not limited to meat, as milk and eggs were included with red meat, processed meat, and poultry. Furthermore, diet was ascertained via a small 13-item food frequency questionnaire, thus, the authors could not adjust for total energy intake. In a 1993 study, Giovannucci and colleagues [7] reported that high intake of red meat fat was associated with a greater than two-fold risk of advanced prostate cancer (RR = 2.64, 95% CI: 1.21-5.77). In contrast, in a recent analysis of the European Prospective Investigation into Cancer and Nutrition (EPIC) cohort, Crowe et al. [16] observed inverse associations of 0.94, 0.83, and 0.84 for fat from red and processed meat and total prostate cancer, advanced prostate cancer, and high-grade prostate cancer, respectively.

Investigations of cooking practices, meat doneness, and dietary mutagens have not produced patterns of associations consistent with increasing the risk of prostate cancer, although the available epidemiologic data are limited to few studies. Barbequed and pan-fried meat has been associated inversely with prostate cancer in three large prospective studies [24,29,41]. However, in a sub-group analysis of the aforementioned NIH-AARP cohort, significant positive associations were reported for grilled/barbequed meat and total and advanced prostate cancer but a non-significant inverse association was observed for fatal prostate cancer [26]. In the same study, no associations were observed for pan-fried, microwaved, or broiled meat and total, advanced, or fatal prostate cancer [26]. The relationship between doneness of meat intake and prostate cancer has been inconsistent as two studies reported significant positive associations between consumption of well and very well done meat and prostate cancer risk [24,28] , and two studies observed no associations for well or very well done meat and prostate cancer [17,26]. Few studies have evaluated dietary mutagens and prostate cancer, and no statistically significant associations have been observed for total mutagenic activity, 2-amino-3,8-dimethylimidazo[4,5-f]quinoxaline (MeIQx), 2-amino-3,4,8-trimethylimidazo[4,5-f]quinoxaline (DiMeIQx), or Benzo[a]pyrene (BaP), with most RRs slightly above or below the null value [24,28], although a marginally significant RR of 1.28 (95% CI: 1.00-1.65) for BaP has been reported for advanced prostate cancer [26]. A statistically significant positive association between 2-amino-1-methyl-6-phenylimidazo[4,5-b]pyridine (PhIP) and total prostate cancer and incident prostate cancer was reported in one study [28], although null or inverse associations were observed between the highest quintile of PhIP and total, advanced, and fatal prostate cancer in another study [26]. The highest quintiles of heme iron, nitrite from meat, and nitrate from meat were associated positively and significantly with advanced prostate cancer among participants in the NIH-AARP cohort, however, no significant associations were observed for total or fatal prostate cancer with the exception of heme iron and total prostate cancer [26]. Additional studies are necessary to fully evaluate any potential associations between consumption preferences, dietary mutagens, heme iron, nitrite/nitrate and prostate cancer.

In the current quantitative assessment of red meat and processed meat intake and prostate cancer, data from prospective studies were analyzed, with the majority of data coming from large cohorts published within the past eight years. Collectively, most meta-analysis summary associations for red and processed meat were null, or just above or below the null value, and not statistically significant. Summary results for processed meat were weakly elevated; however, the association across the more recently published studies that adjusted for key factors was attenuated and not statistically significant. Furthermore, there was evidence of publication bias across the cohort studies of processed meat. In conclusion, the results of this meta-analysis of prospective studies do not support an independent positive association between intake of red meat or processed meat and prostate cancer.

## Conflicts of interests

The authors received partial funding support from the Cattlemen's Beef Board, through the National Cattlemen's Beef Association (NCBA). NCBA did not contribute to the writing, analysis, or interpretation of research findings. All data included in this manuscript were extracted from peer-reviewed published literature.

## Authors' contributions

DDA contributed to the methodological design, writing, analysis, and completion of the manuscript; PJM contributed to the writing and technical overview; CAC contributed to the database management and editorial review; BS contributed to the data extraction and technical editing. All authors read and approved the final manuscript
